# Cancer-testis antigen lactate dehydrogenase C4 as a novel biomarker of male infertility and cancer

**DOI:** 10.3389/fonc.2022.936767

**Published:** 2022-11-02

**Authors:** Jing Wu, Yan Chen, Yingying Lin, Fenghua Lan, Zhaolei Cui

**Affiliations:** ^1^ Laboratory of Biochemistry and Molecular Biology Research, Fujian Key Laboratory of Advanced Technology for Cancer Screening and Early Diagnosis, Department of Clinical Laboratory, Clinical Oncology School of Fujian Medical University, Fujian Cancer Hospital, Fuzhou, China; ^2^ Fuzong Clinical College, Fujian Medical University, Fuzhou, China

**Keywords:** cancer, infertility, cancer-testis antigen, lactate dehydrogenase C4, biomarker

## Abstract

A unique lactate dehydrogenase (LDH) isoenzyme designated as lactate dehydrogenase C4 (LDH-C4) is found in mammalian mature testis and spermatozoa. Thus far, LDH-C4 has been well studied with regard to its gene and amino acid sequences, structure, biological properties, and peptide synthesis. Accumulating evidence has shown that LDH-C4 is closely related to spermatic energy metabolism and plays a critical role in sperm motility, capacitation, and fertilization. Defects in the catalytic activity of LDH-C4 are key to pathophysiological abnormalities underlying infertility. LDH-C4 was originally thought to be present only in mature testis and spermatozoa; however, recent studies have implicated LDH-C4 as a cancer-testis antigen (CTA), owing to its aberrant transcription in a broad spectrum of human neoplasms. This review highlights the recent findings on LDH-C4 with particular emphasis on its role in male infertility and tumors.

## Introduction

Lactate dehydrogenase (LDH) isozymes are widely distributed throughout mammalian tissues and catalyze the interconversion of pyruvate and lactate, representing the last step of anaerobic glycolysis (1, 2). In humans, two subunits of LDH were initially identified and designated as A and B, respectively. Functionally, the A- and B-subunits can assemble into five forms of tetrameric isoenzymes (LDH-1 to LDH-5). In the 1960s, the sixth LDH isoenzyme was identified from the human mature testis and originally named LDH-X (3, 4). Gradual research confirmed LDH-X as a tetrameric protein comprising four identical C-subunits. Thus, it was endowed with another name—LDH-C4 or LDHC (5).

LDH-C4 has been well studied in mammals, particularly in humans and murine models. Interestingly, the catalytic properties of the LDH isoenzymes are reflected through their varying compositions and expression patterns across organs and tissues. For instance, LDHA is abundant in anaerobic tissues including the skeletal muscle, wherein oxygen deficiency during exercise necessitates glycolysis to support the metabolic needs (6). LDHB is predominately found in the brain and heart and plays a critical role in maintaining aerobic metabolism by converting lactate from anaerobic glycolysis (7). In particular, LDH-C4 is mainly present in spermatids and spermatozoa within the mature testis (3–5). LDH-C4 is associated with glucose and plays an essential role in maintaining ATP production in the spermatozoa (8, 9).

Cancer-testis antigen (CTA) belongs to a group of cancer-associated antigens with normal expression in the adult testis but aberrant levels in several types of cancers, particularly in advanced stages exhibiting stem cell–like characteristics (10). Growing evidence suggests that LDH-C4 is a key enzyme for sperm function, and its abnormal activity or function contributes to male infertility (8, 9, 11–14). Moreover, the LDH-C4 isoenzyme is a molecular CTA with aberrant expression in several human cancers (15, 16). In this review, we have focused on gene regulation, tissue distribution, and molecular characteristics of LDH-C4 with particular emphasis on the roles of LDH-C4 in male infertility and tumors.

## *LDHC* gene expression and regulation

### *LDHC* gene and its regulation

**Figure 1:**
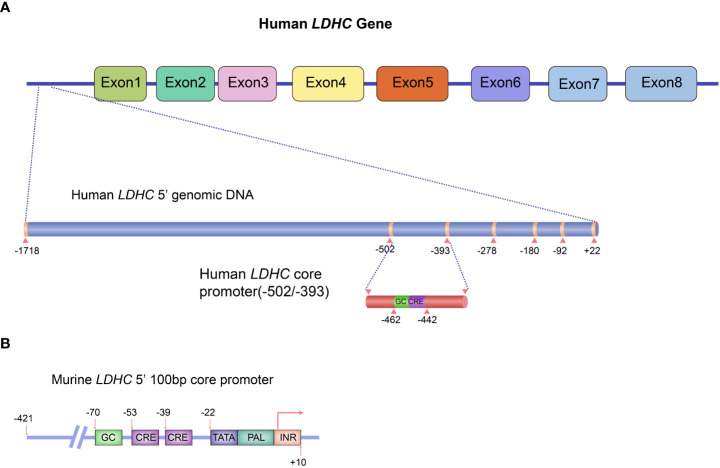
Schematic diagram of *LDHC* gene structure and the regulatory elements for gene expression. **(A)** Human *LDHC* gene structure and its cis-regulatory elements and known trans-acting transcriptional regulators. **(B)** The 100-bp core promoter murine LDHC and its elements. The schematic diagram was plotted in line with the published studies (17, 19).

In humans, expression of the C-subunit is under the control of the *LDHC* gene located at 11p15.3-15.5. Human *LDH*C yields two transcripts with a full-length mRNA of 2,089 bp for transcript variant 1 (NM_002301.5) and 2,035 bp for transcript variant 2 (NM_017448.5). Both variants encode a protein of 332 amino acids. The coding region of *LDHC* comprises seven exons (exons 2~8), whereby exon 1 plays no functional role in coding polypeptides but functions as a transcriptional regulator. The promoter sequences of human *LDHC* have been identified. The 5′-untranslated region (UTR) possesses several ubiquitous cis-acting elements, including one palindrome (PAL), one GC-box, one TATA-box, and two putative CCAAT elements (17). Specifically, the NF-I (nuclear factor I) protein binding site in PAL is adjacent to the TATA box, whereas three DNase I hypersensitive sites are located upstream of the CCAAT elements (17, 18). The NF-I proteins play a functional regulatory role in *LDHC* expression by binding to NF-I–specific sequences of PAL (18). A 110-bp core promoter region comprising a conserved GC-box and two cAMP-responsive element (CRE) binding sites has been identified (19). The GC-box and CRE sites adjoin each other and are all located upstream of the TATA-box (19). Both GC-box and CRE sites are essential for basal *LDHC* transcription (19). Mutations in GC-box or CRE sites reduce 73% and 74% of the total promoter activity, respectively (17). Further studies have demonstrated that a 60-bp sequence in the core promoter region is sufficient to drive robust transcription of *LDHC* in testis (20, 21). PAL may not be essential for *LDHC* transcription (17), but a mutation in the 31-bp PAL sequence in the core promoter region can abolish *LDHC* transcription in mice (22). Other transcription factors, including MYBL1, also play a critical role in regulating *LDHC* expression. A study highlighted that *LDHC* transcription was lost in 21-day-old testes of MYBL1 mutant mice (23). In fact, MYBL1 activates the transcription of *LDHC* in mice by interacting with the proteins that bind to CRE cis-element in the promoter region (23). The gene structure of *LDHC* includes its cis-regulatory elements and known trans-acting transcriptional regulators as shown in **Figures 1A, B**.

### Molecular characteristics of LDH-C4 and its expressions in somatic tissues/cells

LDH-C4 reveals distinct enzymatic, physicochemical, and immunological properties that differ significantly from other LDH isoenzymes (5, 24–27). Intriguingly, although human and mouse LDH-C4 share high–amino acid sequence similarity, some biochemical properties are substantially different. As exemplified by a previous study, mouse LDH-C4 is more thermostable than other LDH isozymes, which retain most of the biological activity after incubation at 65°C for 30 min (25); in comparison, the thermostability of human LDH-C4 is inferior. In addition, the protein distribution of LDH-C4 differs in mature sperms of humans versus mice: LDH-C4 mainly concentrates in the neck segment of the human mature sperms, whereas it is most abundant in the sperm principal piece of mice (5, 28).

Originally, the expression of LDH-C4 was thought to be highly tissue-specific and restricted to mature testis and germ cells, including the spermatocytes, spermatids, and spermatozoa (3, 4, 29). Goldberg et al. confirm that the *LDHC* gene is expressed first in leptotene-zygotene spermatocytes, with the highest mRNA expression in spermatids (5). LDH-C4 is responsible for more than 80% of the total LDH activity in mouse spermatozoa. It is absent in pre-pubertal testes and, thus, can be used as a prospective biomarker for germinal epithelium activity (12, 14, 30). However, the subcellular distribution of LDH-C4 in germ cells especially in spermatozoa is complex. Immunohistochemical studies have demonstrated mouse LDH-C4 in the cytoplasm of spermatocytes or spermatids as well as in the principal and middle pieces of the sperm tail (5, 29). In addition, LDH-C4 is present on the surface of human and murine spermatozoa and is closely related to its immune antiserum binding function (29, 31). In particular, LDH-C4 is present in the matrix of sperm-type mitochondria and forms the mitochondrial sheath in spermatocytes, spermatids, and spermatozoa (29, 32, 33); however, cross-contamination during indirect detection cannot be ruled out, and the role of LDH-C4 in the formation of the mitochondrial sheath in male germ cells remains controversial. Moreover, experiments using the specific antibodies also positioned human LDH-C4 in the post-acrosome area, the neck, and the mid-piece (34). Utilizing the histochemical staining and immunofluorescence techniques, LDH-C4’s localization in human spermatozoa shows strong signals in the neck region (with a high concentration of mitochondria), but these are weak in the middle piece (28).

Thus, LDH-C4 is not strictly testis-specific, as evidenced by its presence in other non-testicular tissues or cells. Coonrod et al. have ascertained the transcription of *LDHC* in oocytes and early embryos; LDH-C4 protein is found in the cortex of oocytes, ova, zygotes, and embryonic blastomeres (27). Although no LDH-C4 activity has been detected in oocytes and the *LDHC-*null female mouse is fertile (12, 27), it does not rule out the fact that LDH-C4 may play a role in oogenesis or ovum function. Recent studies suggest that *LDHC* is transcribed in the somatic cells of the plateau pikas, including the liver, heart, lung, kidney, brain, skeletal muscle, and testis (35). The subcellular distribution of LDH-C4 only appears in the cytosol of somatic cells, whereas it is present in both the cytoplasm and mitochondria of the germ cells in the testis (35).

According to the definition of CTA and the specificity of *LDHC*/LDH-C4 expression in somatic cells, its expression in the peripheral blood of normal subjects is not expected. Interestingly, *LDH*C expression can be detected in the peripheral blood of some healthy person who currently have no apparent clinical abnormalities (35–38). We hypothesize that these healthy individuals with positive serum *LDHC* expression are at high risk of developing tumors or are at early stages of *in situ* tumor or carcinoma development that has not been clinically diagnosed. The above findings shed new light on the functional role of *LDHC*/LDH-C4 in mammals and prompt a host of interesting questions that need further investigations.

## *LDHC*/LDH-C4 and male infertility

### *LDHC*/LDH-C4 are involved in the metabolic pathways in spermatozoa

Gene expression of *LDHA*, *LDHB*, and *LDHC* occurs during the differentiation of germ cells during spermatogenesis (39). *LDHA* is present in pachytene spermatocytes, whereas *LDHB* is typically expressed in Sertoli and spermatogonial cells (39). The synthesis of the *LDHC* gene in testis takes place exclusively during meiosis and spermiogenesis, beginning in preleptotene or leptotene spermatocytes, and reaching its peak in pachytene spermatocytes, spermatids, and spermatozoa (3–5, 28, 29, 33); most LDH activity in male germ cells is attributed to that of LDH-C4 (5). Several investigations have documented the critical role of LDH-C4 in sperm motility, adenosine triphosphate (ATP) production, capacitation, and fertilization (5, 8, 9, 12, 13).

Sperm motility requires high levels of ATP with 70% being utilized for the movement of the flagella alone (40, 41). Two metabolic pathways for energy production operate in the mammalian spermatozoa: oxidative phosphorylation (OXPHOS) and glycolysis (42). OXPHOS takes place mainly in the mitochondria of spermatozoa, whereas glycolysis occurs largely in the head and principal piece (42). Because LDH-C4 is involved in both metabolic pathways and accounts for at least 80% of the total LDH activity in spermatozoa (5, 8, 9, 12), abnormal LDH-C4 function may exert a substantial impact on ATP production, which further weakens sperm function, ultimately leading to sterility. A study revealed that spermatic *LDHC* levels in normal donors are significantly higher relative to those in infertile patients with impaired sperm motility through qRT-PCR analyses, thereby suggesting *LDHC*’s involvement in the motility of spermatozoa (43). The *LDHC* knockout and functional studies based on both mating and preliminary sperm function experiments have ascertained the role of LDH-C4 in the maintenance of male fertility. Although the spermatic morphology or density appears normal, *LDHC* (−/−) male mice are sub-fertile, and the fertility is severely compromised due to a rapid reduction in the level of ATP and motility relative to the wild-type mice. *LDHC* (−/−) sperm does not acquire hyperactivated motility and cannot penetrate the zona pellucida *in vitro*, thus failing to undergo the phosphorylation event characteristic of capacitation (12). Interestingly, the dependence on LDH-C4 for fertilization differs markedly among mice of different genetic backgrounds. As exemplified by the study, *LDHC*-null C57BL/6 (B6) male mice are sub-fertile and the ATP content is moderately attenuated, whereas 129S6 male mice are infertile with drastically reduced ATP levels in spermatozoa (9). Another study further interpreted this interesting phenomenon and found that exogenous LDHA rescued sperm function in *LDHC*-deficient B6 mice (44). These results strongly suggest that *LDHA* is responsible for some or most of the LDH activity in *LDHC*-null sperm. However, on the basis of the current evidence, sperm contains substantially more LDH-C4 than what is required to maintain normal fertility (5). Thus, the function of LDHC if LDHA alone can provide the terminal reaction of glycolysis remains elusive. Whether any functional redundancy among LDH isozymes exists in sperm needs to be investigated in the future.

LDH-C4 regulates multiple signaling pathways in response to sperm capacitation and acrosome reaction (AR). However, the detailed mechanism remains poorly understood. It is suggested that the cAMP/protein kinase A (PKA) pathway depends on the ATP produced from glycolysis and further activates the signaling cascade for protein tyrosine phosphorylation (PTP) during capacitation (45). Upon regulation of glycolysis for ATP production, LDH-C4 impacts sperm capacitation through the cAMP/PKA pathway, further resulting in impaired sperm function. Accumulating evidence suggests that *LDHC*-null sperm does not undergo the phosphorylation event characteristic of capacitation, owing to reduced ATP levels in spermatozoa (12). Moreover, when LDH-C4 activity is selectively blocked using its inhibitor, PTP levels during capacitation are also attenuated (45). AR is a well-controlled exocytosis process that requires the participation of redox activity and several protein kinases. LDH-C4 can maintain the redox status in capacitated spermatozoa and participate in the cAMP/PKA pathway necessary for AR (46). LDH-C4 concentration is correlated with the acrosomal contents, suggesting its close association with infertility caused due to abnormal acrosomes (47).

### LDH-C4–specific antibodies and immune-infertility

LDH-C4 is a potent anti-fertility target for developing vaccines for immunocontraception. Notwithstanding, evidence for the notion that LDH-C4–specific antibodies induced in the female reproductive tract are targeted toward spermatozoa to block fertilization is present (24). The anti-sperm LDH-C4 (IgG) levels in 177 infertile patients were tested by ELISA, and 54 subjects were found to be anti–LDH-C4 IgG positive; within the infertile group, the positivity rates among male and female patients were 31.46% and 29.55%, respectively, implying that the presence of anti–LDH-C4 IgG likely indicates immuno-infertility (48). Contraceptive “DNA vaccines” for LDH-C4 have been synthesized, and its immuno-contraceptive effects on mice have been evaluated (49). As expected, the antibody titers were measurable in the serum of vaccinated male mice and the reproductive tract secretions of vaccinated female mice. The DNA vaccines manifest strong suppression of fertilization in both treated male and female mice (49). Goldberg et al. have also presented similar results, whereby a declining birth rate was observed in LDH-C4–immunized male baboons (24). Although the immunized animals could recover their fertility potential after a period and showed no autoimmune diseases, the induction with high levels of anti–LDH-C4 serum potentially causes male infertility (49).

### LDH-C4 and environment-related male sterility

In particular, some studies have also raised concerns about the adverse effects of environmental factors on LDH-C4 function. Continuous lighting reduces total LDH and the LDH-C4 activity in rat sperms (50). Similarly, radiation impairs the functional capacity of LDH-C4 by unfolding or dissociating the tetramers and further destroying the secondary and tertiary structures of its C-subunit (51). Constant exposure to chromium (VI) results in a visible disruption of germ cell arrangement near the walls of the seminiferous tubules in chromium (VI)–exposed rats. A constant decrease in the concentration of LDH-C4 in seminal plasma further contributes to diminished reproductive function or infertility (52). Some chemicals, including dibromoacetonitrile (DBAN), tributyltin (TBT), and gossypol, also exert substantial impacts on LDH-C4 activity and other spermatic parameters (16, 53–55). DBAN can induce oxidative stress in the testis and decrease nearly 46% of the testicular activity of LDH-C4 in male mice (53). TBT exerts spermatotoxic effects, resulting in increased sperm abnormalities and a decline in sperm count and quality (54). Some studies, however, also propose opposite views. These suggest that LDH-C4 can be utilized as a target of gossypol for developing contraceptive drugs, as long-term treatment with gossypol or its analogs would result in complete atrophy of the seminiferous epithelium and weaken the fertility potential (55). *LDHC* is upregulated in cases of metastatic lung adenocarcinoma (LUAD). The average transcript level of *LDHC* is higher among male patients or smokers as compared with female patients or non-smokers, respectively. *LDHC* is a putative oncogene responsible for smoking-related lung adenocarcinoma, particularly in male patients with pleural effusions, indicating that smoking to some extent contributes to altered LDH-C4 levels in male patients (56).

## *LDHC*/LDH-C4 and tumors

### Pan-cancer expression of LDH-C4

*LDHC* has been detected in various tumor types with varying levels. For instance, the *LDHC* positivity rate reaches 100% (18/18) in lung adenocarcinoma (LUAD), 76% in high-grade serous ovarian carcinomas (HGSC), and 44% in melanoma (56, 57). Koslowski et al. (15) confirm the expression of *LDHC* mRNA in all tested tumor types; the corresponding reported frequencies are as follows: melanoma (7/16), breast (7/20), colon (3/20), prostate (3/8), lung (8/17), renal (4/7), ovarian (3/7), thyroid (1/4), and cervical cancers (5/6); melanoma (5/8) and lung cancer cell lines (2/6). Another study confirms the positivity rate of *LDHC* at 25% in non–small cell lung cancer (NSCLC) samples (including 102 tumor specimens and seven cell lines) (58). Immunohistochemistry (IHC) of LUAD tissues demonstrates the presence of LDH-C4 in 81.8% of samples (72/88), whereas it was absent in the adjacent normal tissues (59). However, Atanackovic et al. have detected the expression of CTAs in 51 cases of head and neck squamous cell carcinoma (HNSCC) and report no positive expression of *LDHC* mRNA (60). Intriguingly, Chen et al. suggest that *LDHC* mRNA levels are significantly downregulated in osteosarcoma samples (38).

The expression of *LDHC*/LDH-C4 is also altered in genitourinary cancers. Hua et al. have evaluated *LDHC* expression levels in 18 pairs of frozen samples from renal cell carcinoma (RCC) patients (18 cancers and 18 corresponding adjacent tissues) by RT-qPCR. Aberrant *LDHC* expression was observed in 22.2% (4/18) RCC tissues, whereas it was absent in all adjacent tissue specimens. LDH-C4 protein is expressed in RCC tissues (27/133), evidenced by IHC analysis (59). Moreover, *LDHC* mRNA and protein levels were higher in CAKI-2 cells relative to CAKI-1 RCC cells; the HK-2 renal tubular epithelial cells show a low level of *LDHC* expression (59). *LDHC* is also specifically expressed in spermatocytic tumors during the prophase of meiosis (61) as well as in type 2 testicular germ cell tumors (TGCTs) (62).

**Figure 2:**
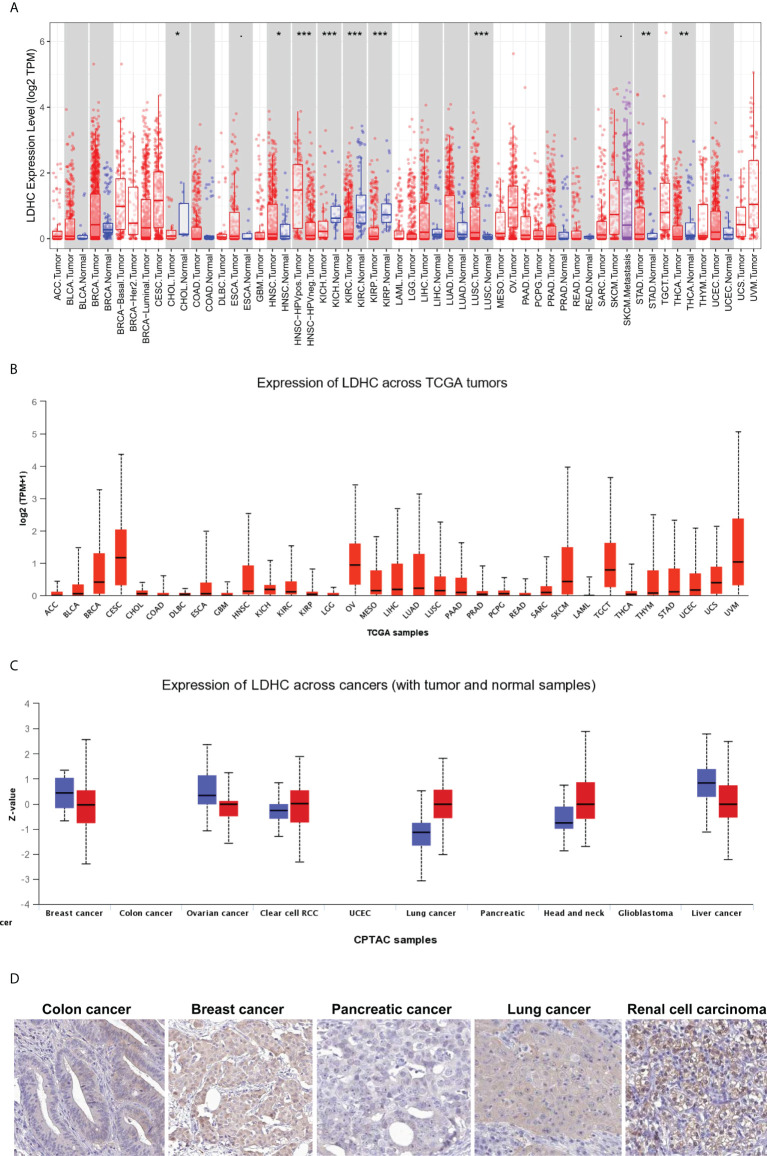
Pan-cancer expression of LDHC/LDH-C4 in public databases. Pan-cancer *LDHC* expression in **(A)** TIMER and **(B)** UALCAN databases. **(C)** Pan-cancer expression analysis of LDH-C4 in the UALCAN database. **(D)** IHC analysis of pan-cancer LDH-C4 using the HPA database. ACC, adrenocortical carcinoma; BLCA, bladder urothelial carcinoma; BRCA, breast invasive carcinoma; CESC, cervical squamous cell carcinoma and endocervical adenocarcinoma; CHOL, cholangio carcinoma; COAD, colon adenocarcinoma; DLBC, lymphoid neoplasm diffuse large B-cell lymphoma; ESCA, esophageal carcinoma; GBM, glioblastoma multiforme; HNSCC, head and neck squamous cell carcinoma; KICH, kidney chromophobe; KIRC, kidney renal clear cell carcinoma; KIRP, kidney renal papillary cell carcinoma; LAML, acute myeloid leukemia; LGG, brain lower-grade glioma; LIHC, liver hepatocellular carcinoma; LUCA, lung cancer; LUAD, lung adenocarcinoma; LUSC, lung squamous cell carcinoma; MESO, mesothelioma; OV, ovarian serous cystadenocarcinoma; PAAD, pancreatic adenocarcinoma; PCPG, pheochromocytoma and paraganglioma; PRAD, prostate adenocarcinoma; READ, rectum adenocarcinoma; RCC, renal cell carcinoma; SARC, sarcoma; SKCM, skin cutaneous melanoma; STAD, stomach adenocarcinoma; TGCT, testicular germ cell tumors; THCA, thyroid carcinoma; THYM, thymoma; UCEC, uterine corpus endometrial carcinoma; UCS, uterine carcinosarcoma; UVM, uveal melanoma. *P < 0.05, **P < 0.01, and ***P < 0.001.

The pan-cancer expression of *LDHC*/LDH-C4 has been assessed, and the results of IHC based on high-throughput tissue microarray analyses suggest the highest positivity rate of LDH-C4 in LUAD (96.7%) (63), followed by breast cancer (BC) (91.55%) (36), and hepatocellular carcinoma (HCC) (55.84%) (37). *LDHC* is expressed in serum and serum exosomes of tumor patients, with serum *LDHC* positivity rates of 91.66%, 68%, and 65% in BC (36), HCC (37), and LUAD (63), respectively. Serum-sourced exosomal *LDHC* in BC and HCC also yield comparable positivity rates as those in the serum (36, 37). The pan-cancer expression of *LDHC*/LDH-C4 based on TIMER (64) and UALCAN (65) suggests high levels in cervical squamous cell carcinoma and endocervical adenocarcinoma (CESC), ovarian serous cystadenocarcinoma (OV), uveal melanoma (UVM), TGCTs, skin cutaneous melanoma (SKCM), HNSCC, and BC (**Figures 2A, B**). At the protein level, LDH-C4 expression is high in lung cancer (LUCA), RCC, and HNSCC relative to paired normal controls (**Figure 2C**). LDH-C4 has been detected by IHC in CRC, BC, prostate cancer (PC), LUCA, and RCC with corresponding positivity rates of 50.00%, 22.22%, 25.00%, 25.00%, and 90.91% (**Figure 2D**), respectively, in the Human Protein Atlas (HPA) database (https://www.proteinatlas.org/about/licence). **Table 1** summarizes *LDHC*/LDH-C4 expression and functions in human cancer types based on published studies.

**Table 1:** Expression status of *LDHC*/LDH-C4 in human tumors.

Tumors	Expressions/Clinical Utility/Biological Functions	Biomarker for Disease	Reference
Pan-cancer, melanoma, and lung cancer cell lines	Frequencies of *LDHC* in pan-cancer: melanoma (7/16), breast (7/20), colon (3/20), prostate (3/8), lung (8/17), kidney (4/7), ovary (3/7), thyroid (1/4), and cervix (5/6); melanoma cell lines (5/8) and lung cancer cell lines (2/6); *LDHC* activation was neither mediated by gene promotor demethylation nor induced by hypoxia.	Biomarker	(15)
Melanoma cell lines (A375M and C81-61)	It was found that CpG island hypomethylation as well as transcription factors Sp1 and CREB played a major role in *LDHC* transcription.	/	(19)
Breast cancer (BC)	It showed 91.66% and 87.50% of serum and exosomal *LDHC*-positive cases, respectively, and 91.55% of LDH-C4–positive patients. *LDHC*/LDH-C4 exhibited high discrimination power in diagnosis, efficacy evaluation, relapse monitoring, and prognosis prediction for BC.	Prognostic and diagnostic biomarker	(36)
Hepatocellular carcinoma (HCC)	It showed that 68% and 60% of serum and exosomal *LDHC*-positive cases, respectively, were reported in HCC; patients with high LDH-C4 expression in HCC tissues accounted for 55.84%. *LDHC*/LDH-C4 could be used as a useful biomarker for early diagnosis, efficacy evaluation, relapse monitoring, and prognosis prediction for HCC.	Prognostic and diagnostic biomarker	(37)
Lung adenocarcinoma (LUAD)	*LDHC* is a candidate oncogene in the carcinogenesis of smoking-related LUAD. LDH-C4 was positive in 81.8% of LUAD samples (72/88), whereas no LDH-C4 was found in any adjacent tissues; LDHC induced the EMT and the expression of MMP-2 and MMP-9, so as to achieve xenograft tumor growth and metastatic potential by the activation of the PI3K/Akt pathway both *in vitro* and *in vivo*. LDHC is a prognostic biomarker in LUAD	Biomarker Prognostic biomarker	(56) (63, 66)
Non–small cell lung cancer (NSCLC)	*LDHC* yielded a frequency of 25% in NSCLC; *LDHC* activation was independent of genomic hypomethylation.	/	(58)
Head and neck squamous cell carcinoma (HNSCC)	*LDHC* was negative in 51 cases with HNSCC.	/	(60)
Osteosarcoma	*LDHC* mRNA level was significantly downregulated in osteosarcoma samples	Biomarker	(38)
Renal cell carcinoma (RCC)	*LDHC* was abnormally expressed in 22.2% (4/18) RCC tissues, whereas no *LDHC* expression was found in adjacent tissues; a high percentage of RCC tissues (27/133) contained LDH-C4 protein; *LDHC* had a significant correlation with EMT, which could elevate the expression level of MMP-9 and enhance the migratory ability of RCC cells.	Prognostic biomarker	(59)
Spermatocytic tumor	*LDHC* is specifically expressed in spermatocytic seminomas during the prophase of meiosis.	/	(61)
Testicular germ cell tumors	Some cases of testicular germ cell tumors had a relatively high expression of *LDHC*, which may be due to undiagnosed normal testis elements in the tumors.	/	(62)

### *LDHC*/LDH-C4 in cancer: Biological functions and regulatory mechanisms

LDH-C4 plays an oncogenic role in the majority of malignant tumors. However, reports on the detailed mechanism underlying *LDHC*’s involvement in tumor development are scarce. Much of the research has mainly focused on the correlations of LDH-C4 with epithelial–mesenchymal transition (EMT), MMP-2/MMP-9, and PI3K/AKT/GSK-3β signaling pathways. For instance, suppression of endogenous LDH-C4 significantly inhibits the growth and invasion of CAKI-2 cells, whereas overexpression of *LDHC* enhances the migration and invasion of CAKI-2 cells, in addition to inducing EMT in RCC (59). Wang et al. suggest that *LDHC* correlates substantially with EMT and elevates the expression of MMP-9, thus boosting the migratory ability of RCC cells (59). Chen et al. show that lentivirus-mediated *LDHC* overexpression upregulates MMP-2 and MMP-9 levels in A549 and H1299 LUAD cells (66). Exogenous LDH-C4 upregulation increases the expressions of PI3K and the phosphorylation of Akt (Ser473) and GSK-3β (Ser9) in A549 and H1299 cells, whereas the expressions of Akt and GSK-3β remain unchanged. Conversely, the PI3K inhibitor, LY294002, effectively diminishes the levels of phosphorylated Akt/GSK-3β induced by LDH-C4 overexpression in the two cell lines (66). Consequently, upon treatment with LY294002, the migratory and invasive abilities of LDH-C4–overexpressing A549 cells, EMT, and expression of MMP-2 and MMP-9 are attenuated. Correspondingly, LDH-C4 can be induced by EMT and the expression of MMP-2 and MMP-9 to achieve xenograft tumor growth and metastatic potential by the activation of the PI3K/AKT pathway *in vivo* (66). Collectively, these results demonstrate that LDH-C4 promotes malignant biological activities in LUAD cells by triggering the PI3K/AKT/GSK-3β signaling pathway and promoting EMT (66).

### LDH-C4 and tumor energy metabolism

Cancer cells preferentially convert glucose to lactate and obtain energy by aerobic glycolysis, a phenomenon known as the “Warburg effect” (67). LDH is a key metabolic enzyme for aerobic glycolysis that plays a crucial role in the conversion of pyruvate to lactate (68). *LDHC*/LDH-C4 affects the progression of tumor development mainly by influencing glycolysis. Chen et al. (38) confirm the high abundance of biomarkers in the glycolytic pathway in osteosarcoma, including *LDHC* genes and two metabolites (lactate and pyruvate). Lactate concentration reduces significantly following the blocking of endogenous *LDHC* in CAKI-2 RCC cells (59). *LDHC* overexpression results in increased lactate concentration in CAKI-1 RCC cells (59). In addition to its involvement in metabolism in LUAD cells, *LDHC* overexpression in A549 and H1299 cells results in significantly higher lactate concentrations (66). This results in a consistently high ATP concentration in LUAD cells, suggesting LDH-C4’s importance for lactate metabolism in LUAD cells (66).

### LDH-C4—a biomarker for cancer prognosis, diagnosis, and immunotherapy

**Figure 3:**
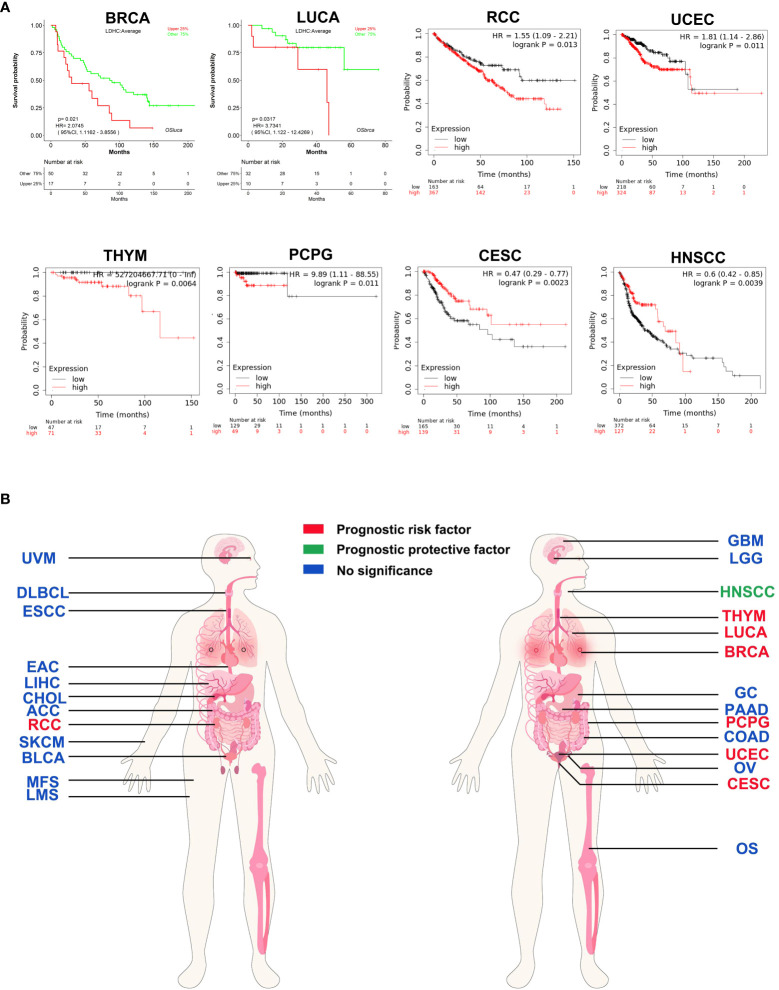
Prognostic pan-cancer analysis of *LDHC* using public databases. **(A)** Survival curves plotted using LOGpc and Kaplan–Meier plotter. **(B)** Pan-cancer view of the prognostic feature of *LDHC* based on the data from LOGpc and Kaplan–Meier plotter. The images of panel **(B)** are from the LOGpc database (https://bioinfo.henu.edu.cn/DatabaseList.jsp). ACC, adrenocortical carcinoma; BLCA, bladder urothelial carcinoma; BRCA, breast invasive carcinoma; CESC, cervical squamous cell carcinoma and endocervical adenocarcinoma; CHOL, cholangio carcinoma; COAD, colon adenocarcinoma; DLBC, lymphoid neoplasm diffuse large B-cell lymphoma; ESCA, esophageal carcinoma; GBM, glioblastoma multiforme; GC, gastric cancer; HNSCC, head and neck squamous cell carcinoma; RCC, renal cell carcinoma; LGG, brain lower-grade glioma; LIHC, liver hepatocellular carcinoma; LUCA, lung cancer; OS, osteosarcoma; OV, ovarian serous cystadenocarcinoma; PAAD, pancreatic adenocarcinoma; PCPG, pheochromocytoma and paraganglioma; SKCM, skin cutaneous melanoma; THYM, thymoma; UCEC, uterine corpus endometrial carcinoma; UVM, uveal melanoma.

LDH-C4 is not cancer-type specific, and most studies suggest that *LDHC*/LDH-C4 can be used as a prognostic indicator of tumors. By analyzing LOGpc (https://bioinfo.henu.edu.cn/DatabaseList.jsp) and Kaplan–Meier plotter (https://kmplot.com/analysis/) databases, *LDHC* was found to be oncogenic in BC, RCC, LUCA, uterine corpus endometrial carcinoma (UCEC), thymoma (THYM), and pheochromocytoma and paraganglioma (PCPG), with high *LDHC* expression being associated with poor prognosis. However, *LDHC* was a protective factor for prognosis in HNSCC and CESC (**Figures 3A, B**). In LUAD (63), LDH-C4 protein was significantly associated with TNM tumor status, and patients with high LDH-C4 expression had a worse prognosis than those with low/negative expression. Similar results were observed in HCC (37) and BC (36). In genitourinary cancer, Hua et al. (59) report that LDH-C4–positive status is significantly associated with advanced tumor stage in RCC (*P* = 0.042). Moreover, positive LDH-C4 expression is significantly associated with an increased risk for poor clinical prognoses in patients with RCC (log-rank *P* = 0.0043) and short OS (59). Why might LDHC be a risk factor for some tumor types but a protective factor for prognoses of other tumor types (HNSCC and CESC) remains unclear? The plausible reasons could be as follows: First, the survival analyses of HNSCC and CESC in Kaplan–Meier plotter were based on limited sample size. Several other cancer types were also included, and the overall combined prognostic effects were more likely to be canceled. Second, because of post-translational modifications of proteins, *LDHC* mRNA levels in mRNA datasets might be inconsistent with the corresponding protein levels, resulting in inconsistent prognostic results in databases.

*LDHC* mRNA has been detected in serum and serum-derived exosomes of patients with BC (36), HCC (37), and LUAD (63), and the source may be related to vesicle encapsulation of tumor cells and release of necrotic tumor cells into the peripheral blood. The AUCs of serum *LDHC* for BC (36), HCC (37), and LUAD (63) reached 0.9587, 0.8382, and 0.8121, respectively, whereas those of serum-sourced *LDHC* for BC, HCC, and LUAD reached 0.9464, 0.9451, and 0.8925, respectively, higher than the corresponding values for serum *LDHC*. These data indicate that *LDHC* is a promising novel non-invasive marker for tumor diagnosis.

Immunotherapy is the fourth most successful treatment strategy for tumors after surgery, chemotherapy, and radiotherapy. Numerous preclinical and clinical investigations focusing on NY-ESO-1 (69), MAGE-A3 (70), and PRAME (71) have shown that CTAs are promising candidates for adoptive T-cell treatment. According to recent research, LDH-C4 may be used as a CTA for targeted immunotherapy (57). The peptide pools (PP1–PP8) comprising 10–11 individual peptides each have been investigated for the immunogenicity of LDH-C4. These are indeed immunogenic; individual peptides within PP2 and PP8 can induce LDH-C4–specific T cell responses in HLA-A*0201 donors. Notably, PP2- and PP8-specific T cells exhibit cytotoxic activity toward BC cells expressing endogenous LDH-C4 within an HLA-A*0201 model. Peptides LDH-C4 amino acid (aa) 41−55 and aa288−303 from PP2 and PP8 elicit a functional cellular immune response. An increase in IFN-γ secretion by CD8^+^ T cells and cancer cell killing of HLA-A*0201/LDH-C4–positive breast cancer cells by LDH-C4 aa41−55– and aa288−303–induced T cells have been documented (57). This study supports the rationale to assess LDH-C4; in particular, the HLA-A*0201 restricted LDH-C4 (aa41–55) and LDH-C4 (aa288–303) peptides (57), as a CTA for targeted immunotherapy. Another study highlights that *LDHC* expression is upregulated in the responder population among patients with melanoma undergoing treatment with anti–PD-1 therapy, suggesting that LDHC is a potential predictive biomarker of response to immune checkpoint inhibitor therapy (72).

## Conclusions and perspectives

LDH-C4 is a critical isoenzyme required for sperm motility, capacitation, and fertilization. As such, it can be employed as an important parameter in evaluating semen quality and male reproductive function. Owing to its status as a CTA gene, *LDHC* has therapeutic potential for immunotherapy against tumors. *LDHC* as a target CTA for immunotherapy has been exploited in BC; consequently, it is necessary to conduct in-depth research for malignant tumors including BC. Furthermore, a better knowledge of *LDHC*’s aberrant transcription profile in cancer cells will shed light on its role in tumor progression. Further investigations are needed to validate the functional involvement of LDH-C4 in the treatment of carcinomas. The expression of *LDHC*/LDH-C4 in somatic cells and peripheral blood of some healthy people other than in tumors and testis necessitates further evaluation. Circulating *LDHC* has the potential to provide new clues for the early diagnosis of tumors, but these data need to be confirmed further.

## Author contributions

ZC, FL, and YL conceived and designed the study. JW and YC wrote the manuscript, and ZC and YL proofread the manuscript. All authors approved the final version of the manuscript.

## Funding

This study was sponsored by the National Natural Science Foundation of China (grant no. 81802631), Provincial Natural Science Fund of Fujian (grant nos. 2021J01439 and 2021J01444), and Joint Funds for the Innovation of Science and Technology, Fujian province (grant nos. 2021Y9221 and 2021Y9222), and Student Innovation Project of Fujian Medical University (grant no. C21157).

## Acknowledgments

We thank the students—Yue Xiao, Lan Wu, Jiajun Lin, Qingyun Wang, and Qiaochu Zhang—from the Fujian Medical University in participation of literature collection. We also thank Bullet Edits Limited for the linguistic editing and proofreading of the manuscript.

## Conflict of interest

The authors declare that the research was conducted in the absence of any commercial or financial relationships that could be construed as a potential conflict of interest.

## Publisher’s note

All claims expressed in this article are solely those of the authors and do not necessarily represent those of their affiliated organizations, or those of the publisher, the editors and the reviewers. Any product that may be evaluated in this article, or claim that may be made by its manufacturer, is not guaranteed or endorsed by the publisher.

## Abbreviations

aa, amino acids; ATP, adenosine triphosphate; AR, acrosome reaction; BC, breast cancer; cAMP, cyclic adenosine monophosphate; CTA, cancer-testis antigen; CTC, circulating tumor cell; CRE, cAMP-responsive element; ELISA, enzyme-linked immunosorbent assay; EMT, epithelial–mesenchymal transition; HCC, hepatocellular carcinoma; KO, knockout; HNSCC, head and neck squamous cell carcinoma; LDH, lactate dehydrogenase; LDHC, lactate dehydrogenase C; LDH-C4, lactate dehydrogenase C4; LUAD, lung adenocarcinoma; LUCA, lung cancer; MYBL1, myoblastosis protein L1; NF-I, nuclear factor I; NAD, nicotinamide adenine dinucleotide; NADH, nicotinamide adenine dinucleotide hydrogen; NSCLC, non–small cell lung cancer; OXPHOS, oxidative phosphorylation; PAGE, polyacrylamide gel electrophoresis; PAL, palindrome; PTP, protein tyrosine phosphorylation; PKA, protein kinase A; qRT-PCR, quantitative real-time polymerase chain reaction; RCC, renal cell carcinoma; ROS, reactive oxygen species; Sp1, specificity protein; TBT, tributyltin; UTR, untranslated region.
